# Isolated pediatric transolecranon fracture‐dislocation of the elbow managed nonoperatively: A case report and review of literature

**DOI:** 10.1002/ccr3.2268

**Published:** 2019-06-17

**Authors:** Abdul Rehman Arain, Stefanos Haddad, Matthew Anderson, Hamza Murtaza, Andrew Rosenbaum

**Affiliations:** ^1^ Albany Medical Center Albany New York

**Keywords:** pediatric elbow fracture‐dislocation, pediatric olecranon fracture‐dislocation, pediatric orthopedic, transolecranon fracture‐dislocation

## Abstract

We report on the sixth case of an isolated pediatric transolecranon fracture‐dislocation, and the first case utilizing nonoperative management in a cast following closed reduction resulting in an excellent outcome. Our case provides support for nonoperative management of these rare injuries, especially when surgery is not practical or desirable.

## 1 INTRODUCTION

Transolecranon fracture‐dislocations are well recognized in adults but rarely occur in children. To our knowledge, only seven cases of transolecranon fracture variants have been reported in literature, only five of which are isolated olecranon fracture‐dislocations. All five patients were managed operatively with good outcomes. We report on the sixth case of an isolated olecranon fracture‐dislocation, and the first case utilizing nonoperative management in a cast following closed reduction. At 3.5 months, the patient returned to gym class with a 20‐degree flexion contracture, which resolved at 5 months.

Anterior fracture‐dislocations of the ulnohumeral joint, transolecranon fracture‐dislocations, are relatively uncommon injuries.^1^, ^2^ The injury is the result of a direct blow to the posterior forearm with the elbow at midflexion. It is characterized by a disruption of the ulnohumeral joint, via a fracture through the trochlea while maintaining radioulnar alignment. The sparing of radioulnar alignment is what differentiates the transolecranon fracture‐dislocations from Monteggia variants, with the majority of the capsuloligamentous restraints intact, particularly the annular ligament.^3^

The transolecranon fracture‐dislocation has been relatively well described in adults but there are very few case reports describing the injury and treatment in the pediatric population. The cases described in literature have all been treated surgically, either with plate fixation or tension band constructs.^4^, ^5^ However, many of the described cases necessitated surgery due to concomitant injury, medial epicondyle fracture, or open injury. We report on the sixth case of a “pure” olecranon fracture‐dislocation, and the first case utilizing nonoperative management in a cast following closed reduction.

## 2 CASE REPORT

**Figure 1:**
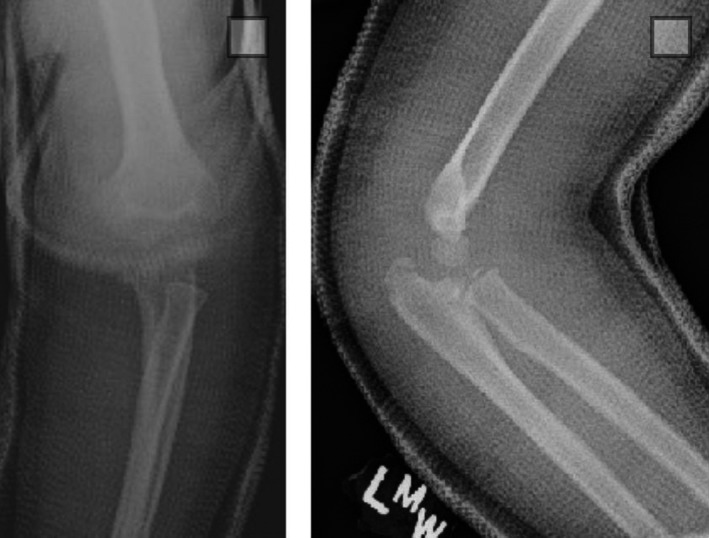
AP and lateral injury films of the elbow showing a transolecranon fracture‐dislocation

**Figure 2:**
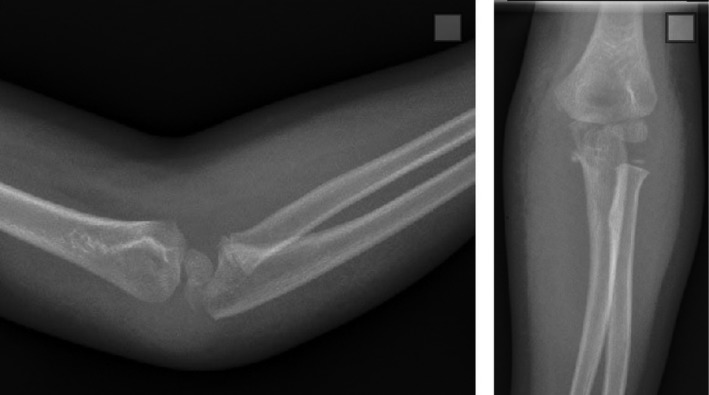
AP and lateral of the elbow after closed reduction and cast application

A 7‐year‐old girl presented to the emergency department with a painful, swollen, and grossly deformed left elbow after a 3‐feet fall in the school playground. Her range of motion at the elbow was restricted by pain, but she maintained full range of motion and sensation at the wrist and fingers. Her radial pulse was palpable and equal to that of the contralateral side. X‐rays of the elbow revealed an anterior and lateral transolecranon elbow fracture‐dislocation without significant comminution (Figure 1). The patient underwent immediate closed reduction utilizing inline traction of the distal forearm, followed by a posterior force on the forearm and an anteriorly directed force on the distal humerus. The patient was immobilized in a long arm cast in neutral rotation and made nonweight bearing (Figure 2). After a thorough discussion, the surgeon of record and the patient's parents agreed on nonoperative management with close observation.

At 1 month, the patient was transitioned to a splint. At 6 weeks, the splint was removed and gentle range of motion was started. At the 3‐month mark, the patient was noted to have full flexion, supination, and pronation. The patient developed flexion contracture of approximately 30° which resolved after a subsequent course of physical therapy. By 5 months, the patient resumed participation in gym class and had full range of motion at the elbow joint. Given the excellent clinical examination, the parents agreed to continue with nonoperative management with regular follow‐ups in clinic spaced 3‐4 months apart.

## 3 DISCUSSION

In adults, the transolecranon fracture‐dislocation has been well described in literature. It is classically described as an osseous lesion of the elbow, where disruption between the olecranon and trochlea notch occurs as the forearm dislocates anteriorly in relation to the trochlea. The collateral ligaments remain intact, and the radial head forms a complex with the distal ulnar fragment.^6^

In the pediatric population, a pure transolecranon fracture pattern is incredibly rare with only five cases reported in the literature (Table 1). Tiemdjo described a classification scheme for anterior transolecranon fracture‐dislocations in children.^7^ It is possible that transolecranon fracture‐dislocations are underreported because they can potentially be misclassified as Monteggia type fracture‐dislocations. Monteggia lesions are defined by a dislocation of the proximal radioulnar joint.^2^ Nonetheless, little data exist as to the optimal treatment for the best clinical outcomes.

**Table 1:** Reported cases for *isolated* transolecranon fracture‐dislocation in the pediatric population^4^, ^5^

Author	# Patients/Gender	Age range	Gender	Treatment	Complications	Implant Removal range	Outcome
Guitton et al (2009)	4	8‐13	M	3 open reduction internal fixation, 1 Tension band construct	1 patient had tension band revised to plate for articular incongruity at 3‐wk	7‐13 mo	Full range of motion at an average of 17‐mo
Butler et al (2012)	1	9	M	K‐wire Tension band construct	None	5‐mo	Hardware removed at 5‐mo, full ROM
Our case (2018)	1	4	F	Closed reduction in cast	30‐degree flexion contracture at 3‐mo f/u	Not Applicable	Full range of motion at 5 mo

Previous cases of pediatric transolecranon fracture‐dislocations have been treated with either a tension band construct or plate fixation. Tension band constructs have been favored by some because unlike adult fractures, pediatric patients typically do not have extensive metaphyseal comminution or an associated coronoid fracture allowing for adequate compression at the fracture site.^5^ Others believe that associated soft tissue injury makes tension band wiring insufficient, and plate and screw fixation preferable.^4^

In addition, associated medial epicondyle fractures may require screw fixation to allow for rigid stability and early motion.^5^ An incarcerated fracture fragment obstructing elbow range of motion would be an indication for operative management.

Optimal treatment of the transolecranon fracture‐dislocation, regardless of age group, begins with the identification of the injury and restoration of the trochlear notch of the ulna. Arguments against nonoperative management focus primarily on the concern that the fracture may displace with casting. Since such limited data exist regarding these injuries in the pediatric population, patients are often treated with surgical management similar to adults. However, this case suggests that nonoperative management is a good option and could be attempted. The remodeling potential of children should not be underestimated. If the alignment of the fracture is near anatomic, there is a high likelihood of a successful clinical outcome. There must be close follow‐up with thorough physical examinations, so the patient can be switched to surgical management if needed.

## 4 CONCLUSION

Transolecranon fracture‐dislocations in the pediatric population rarely occur. Under certain circumstances, good clinical outcomes can be achieved with nonoperative management, utilizing early mobilization and physical therapy. We recommend close follow‐up for a prolonged period of time, even after substantial clinical improvement. We hope future clinicians can utilize our case and find solace in attempting to treat these rare injuries nonoperatively. This may offer a practical advantage in situations where surgery is not possible such as religious objections, parental/patient objections, and resource limitations, and the availability of pediatric orthopedic specialist is limited.

## CONFLICT OF INTEREST

The authors declare that they have no competing interests and nothing to disclose.

Informed Consent: Written informed consent was obtained from this patient authorizing the publication of case details and associated images. The study protocol was approved by the institute's committee on human research.

## AUTHOR CONTRIBUTIONS

ARA, SH, MA, HM, and AR: participated in the diagnosis and treatment of this patient. All authors: contributed to the preparation of this manuscript equally. ARA: the lead author and was additionally responsible for submission and making appropriate edits when needed. All authors: approved of the final manuscript.
